# Long-term survival case of esophageal carcinosarcoma coexisting with alcoholic liver cirrhosis successfully treated by staged operation: A case report

**DOI:** 10.1016/j.ijscr.2021.105946

**Published:** 2021-04-30

**Authors:** Fumihiko Kato, Kazuo Koyanagi, Shiro Sugihara, Tomohiko Nakagawa, Koji Hayashi, Junichi Shintoku

**Affiliations:** a Department of Surgery, Ota Memorial Hospital, 455-1 Oshima-cho, Ota, Gunma 373-8585, Japan; b Department of Pathology, Ota Memorial Hospital, Japan; c Department of Gastroenterological Surgery, Tokai University School of Medicine, 143 Shimokasuya, Isehara, Kanagawa 259-1193, Japan

**Keywords:** Esophageal carcinosarcoma, Staged operation, Liver cirrhosis

## Abstract

**Introduction:**

Patients with esophageal cancers including carcinosarcoma sometimes have underlying liver cirrhosis because of a history of heavy drinking. It is definitely required to determine the appropriate surgical strategy and to manage the patients promptly when performing esophagectomy for the esophageal carcinosarcoma coexisting with alcoholic liver cirrhosis.

**Presentation of case:**

A 56-year-old male patient with a history of chest pain and difficulty swallowing was admitted to our hospital. He had a history of drinking 250 g of alcohol per day. Endoscopy revealed an irregular protruding tumor on the left wall of the lower-third thoracic esophagus. Computed tomography showed a tumor lesion in the lower-third thoracic esophagus; the images also showed irregularities on the surface of the liver, suggestive of coexisting alcoholic liver cirrhosis. The preoperative diagnosis was T3N2M0, Stage III esophageal leiomyosarcoma. In consideration of the underlying alcoholic liver cirrhosis, a staged operation was planned for this patient as a curative treatment. The patient had an uneventful postoperative clinical course and was discharged on the 47th day after the first surgery. Final histopathological diagnosis was T2N0M0, Stage II esophageal carcinosarcoma. The patient is alive without recurrence three years after surgery.

**Discussion:**

This is the first report of long-term survival case of esophageal carcinosarcoma with alcoholic liver cirrhosis that was treated successfully by staged operation.

**Conclusions:**

Despite coexisting with alcoholic liver cirrhosis, staged operation could reduce the surgical invasiveness, so that very good short-term outcome and long-term survival was obtained in the patient with esophageal carcinosarcoma.

## Highlights

• Patients with esophageal carcinosarcoma sometimes have underlying liver cirrhosis because of a history of heavy drinking.
• Postoperative severe complications often occur after esophagectomy in patients with liver cirrhosis.
• Staged operation could reduce the surgical invasiveness and manage these patients.

## 1 Introduction

Esophagectomy is a particularly invasive operation, and postoperative complications are frequent [1]. As esophageal cancer patients often have a history of heavy drinking, they sometimes have underlying liver cirrhosis, which increases the risk of postoperative complications after esophagectomy [2]. Therefore, careful selection of the surgical strategies and strict perioperative management are required in these patients. In the present study, we report a long-term survival case of esophageal carcinosarcoma patients with alcoholic liver cirrhosis who was successfully treated by a staged operation.

This case report is compliant with the Updating Consensus Surgical Case Report (SCARE) guidelines. A complete SCARE checklist was submitted along with the original manuscript [3].

## 2 Presentation of case

A 56-year-old male patient presented with a 3-month history of having suffered from chest pain and swallowing difficulty. His body weight had decreased by 10 kg during this 3-month period. The patient was admitted to our hospital with the diagnosis of esophageal tumor in the lower-third thoracic esophagus made by a general practitioner. The patient had no apparent significant medical history prior to the current illness. However, he had a history of having smoked 1.5 packs of cigarettes a day for 36 years and drinking 250 g of alcohol per day.

**Fig. 1:**
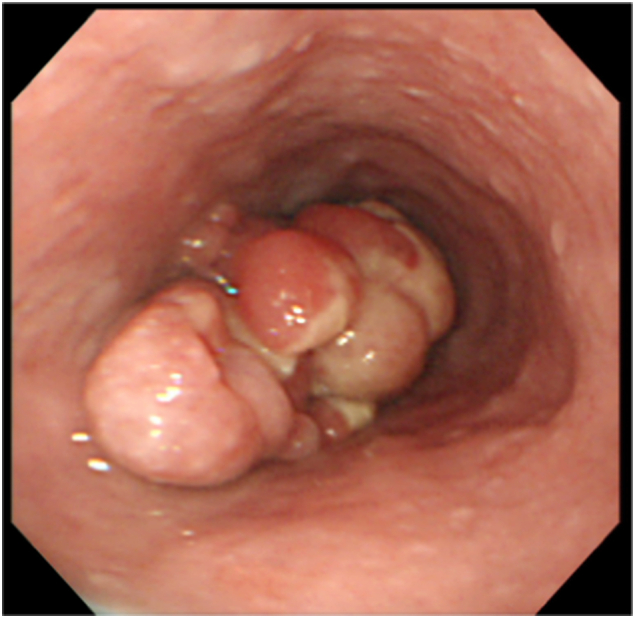
Gastrointestinal endoscopic image. Irregular protruding tumor visualized in the left wall of the lower-third thoracic esophagus.

**Fig. 2:**
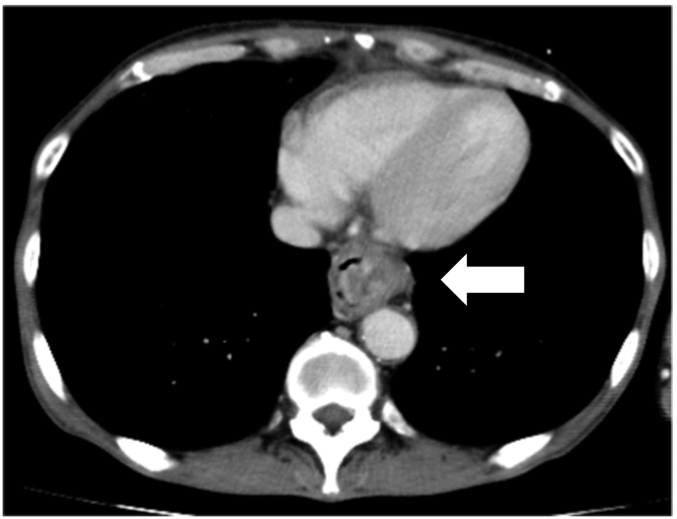
Enhanced computed tomography image. Tumor visualized in the lumen of the lower-third thoracic esophagus (arrow).

**Fig. 3:**
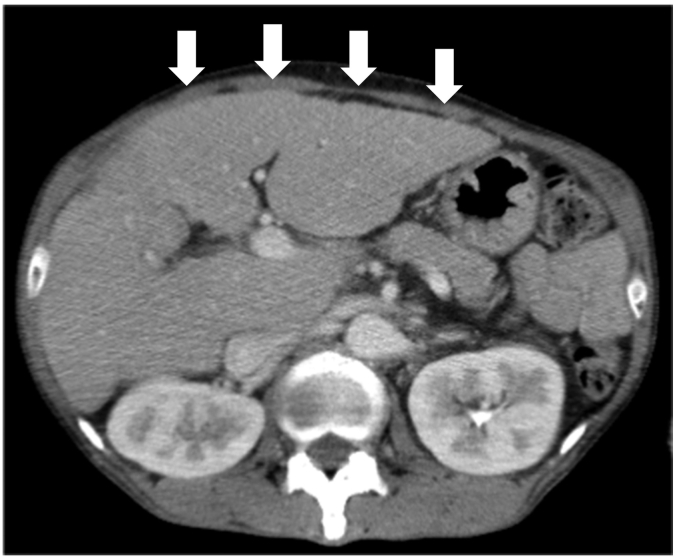
Enhanced computed tomography image. Irregularities are seen on the surface of the liver, suggestive of liver cirrhosis (arrows).

The serum albumin was decreased to 3.0 g/dL (normal range > 3.9 g/dL). Other blood parameters were normal and tests for the hepatitis virus markers were negative. The electrocardiogram and plain chest X-ray revealed no abnormalities. Endoscopy revealed an irregular protruding tumor on the left wall of lower-third thoracic esophagus (Fig. 1). There was no evidence of esophageal varices. Histopathological examination of biopsy specimens obtained from the tumor showed atypical cells arranged in bundles. Immunohistochemistry revealed positive staining of the cells for vimentin and negative staining for desmin, SMA, S-100, and HMB-45. Based on these findings, the tumor was diagnosed as a leiomyosarcoma. Computed tomography revealed a tumor in the lumen of the lower-third thoracic esophagus (Fig. 2). Slightly enlarged lymph nodes were detected around the right recurrent laryngeal nerve and left gastric artery, and were suspected to be metastatic lymph nodes. There were no obvious distant metastases. Irregularities were found on the surface of the liver, suggestive of liver cirrhosis (Fig. 3).

The preoperative diagnosis was esophageal leiomyosarcoma and the tumor stage (according to the eighth edition of the Union for International Cancer Control) was T3N2M0, Stage III. The patient also had Child-Pugh class A alcoholic liver cirrhosis. In consideration of the surgical risk associated with the underlying liver cirrhosis, we planned staged operation for the patient. Resection of the thoracic esophagus, mediastinal lymph node dissection via a right thoracotomy, and cervical esophagostomy were performed in the first-step surgery. The patient was extubated on the 1st postoperative day. Total parenteral nutrition was administered until the next operation. Acute cholecystitis occurred on the 9th postoperative day and percutaneous transhepatic gallbladder drainage was performed. Abdominal lymph node dissection, reconstruction of the gastric tube via the subcutaneous route, cholecystectomy, and jejunostomy were performed in the second-step surgery on the 19th day after the first surgery. The liver was swollen with irregular surface, suggestive of liver cirrhosis. Small amount of ascites was recognized during operation.

**Fig. 4:**
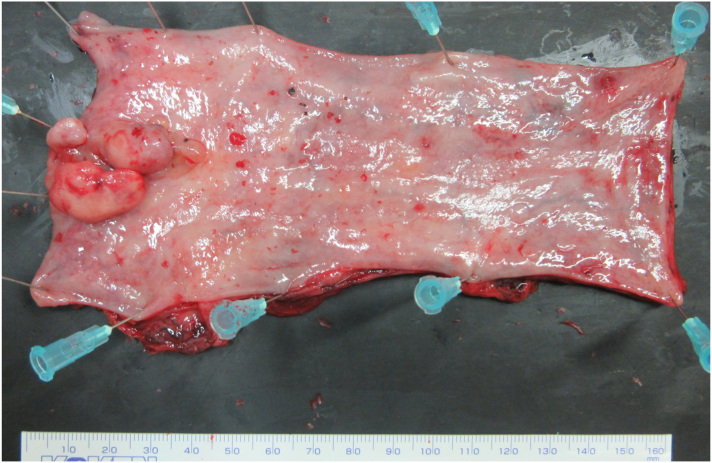
Thoracic esophagus resected at the first surgery. Macroscopic examination of the resected specimen showed an irregular protuberant tumor in the lower-third esophagus. The anal-side surgical margin was diagnosed to be potentially positive for tumor, and was completely resected at the second surgery.

**Fig. 5:**
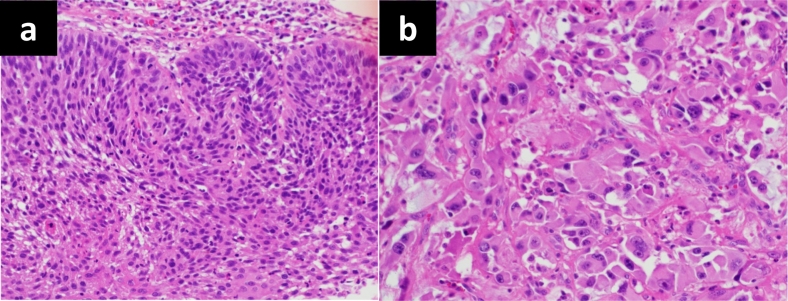
Microscopic appearance of the esophageal tumor stained with hematoxylin and eosin. The resected tumor showed both squamous cell carcinoma arising from the epithelium (5a) and spindle-shaped bizarre giant cells in the stroma (5b) (×200).

The patient had an uneventful postoperative clinical course after the second surgery and was discharged on the 47th day after the first surgery. Macroscopic examination of the resected specimen showed an irregular protruding tumor in the lower-third esophagus. The anal-side surgical margin of the thoracic esophagus resected at the first surgery was diagnosed to be potentially positive for cancer cells (Fig. 4). However, it was resected completely in the second surgery. Histopathological examination showed both squamous cell carcinoma arising from the epithelium (Fig. 5a) and spindle-shaped bizarre giant cells in the stroma (Fig. 5b). The tumor infiltrated the muscular layer of the esophagus, but no metastasis was detected in the harvested lymph nodes. The pathological diagnosis was T2N0M0, Stage II esophageal carcinosarcoma. At 3 years after the operation, the patient remains alive without tumor recurrence.

## 3 Discussion

Esophageal carcinosarcoma is a relatively rare disease, accounting for 0.5%–2.8% of all malignant esophageal tumors [4]. No chemotherapy or radiation therapy has been established yet for esophageal carcinosarcoma, and surgery is the only modality that offers any chance of cure [5]. Five-year survival rate of esophageal carcinosarcoma is poor and was reported to be only 27% [6]. This patient was suspected as having esophageal leiomyosarcoma prior to the surgery and as a case of esophageal carcinosarcoma after the surgery. Although the perioperative risk in this patient was considerably high due to the underlying liver cirrhosis, we have decided to perform esophagectomy as the only available curative therapy. Staged operation could reduce the surgical invasiveness and manage the cirrhosis patient not to occur life-threatening complications after esophagectomy. To the best of our knowledge, this is the first report of long-term survival case of esophageal carcinosarcoma with alcoholic liver cirrhosis that was treated successfully by staged operation.

Postoperative severe complications have often occurred after esophagectomy in patients with liver cirrhosis, so that, during surgery, surgeons must be very careful about bleeding due to portal hypertension, lymphatic leakage, and congestion of the gastric tube. Pleural effusion, ascites, infection, delayed wound healing, liver dysfunction, and renal dysfunction are often encountered as problematic complications after the operation. Anastomotic leakage was seen in a high percentage of patients (13%–83%), and even minor leakages could become fatal [7]. Asti et al. reviewed five retrospective studies involving 157 cirrhotic patients and reported 28 deaths [8]. The main cause of death was liver failure (32%), followed by pneumonia/sepsis (29%) and anastomotic leakage (21%) [8]. Deng et al. reported a meta-analysis of six cohort studies involving 161 patients with and 1265 without liver cirrhosis [9]. Patients with liver cirrhosis had a significantly higher mortality rate than those without cirrhosis after esophagectomy (14.4% vs. 4.7%) (RR = 2.529; 95% CI = [1.480, 4.324]; *P* = 0.001) and had more unfavorable long-term survival (5-year survival rate: 21.1% vs. 32.0%) (RR = 0.715; 95% CI = [0.492, 1.039]; *P* = 0.079) [9]. It is definitely required to reduce the surgical invasiveness in patients with liver cirrhosis in order to avoid postoperative life-threatening complications.

Regarding the association between liver damage and mortality after esophagectomy, Lu et al. reviewed 16 liver cirrhosis patients who underwent esophagectomy and reported mortality rates in Child-Pugh class A, B, and C patients of 10%, 50%, and 100% [10]. Tachibana et al. reported an operative mortality after esophagectomy in 18 patients with Child-Pugh class A and B patients of 16.7% [11]. These studies indicate very high mortality rates associated with esophagectomy in Child-Pugh class B and C cirrhosis patients. On the other hand, patients with Child-Pugh class A liver cirrhosis could be potentially managed by appropriate therapeutic strategies.

Staged operation is often performed in high-risk patients with esophageal tumors to reduce the excessive surgical invasiveness. In the first stage, resection of the thoracic esophagus, mediastinal lymph node dissection, and cervical esophagostomy are performed. In the second stage, abdominal lymph node dissection and reconstruction of the gastric tube or other organs are performed. On the other hand, there have been no previous reports of staged operation for esophageal carcinosarcoma patients with liver cirrhosis, probably because of rarity of esophageal carcinosarcoma and high mortality rate after esophagectomy in patients with liver cirrhosis. However, esophagectomy was the only curative therapeutic modality for esophageal carcinosarcoma and liver damage of the patient was evaluated as Child- Pugh class A. Therefore, we have decided to perform staged esophagectomy with thorough lymph node dissection to ensure the curability and the safety of surgery. Patients with liver cirrhosis are compromised and are easily liable to developing pulmonary complications and anastomotic leakage after esophagectomy [8]. If pulmonary complications and anastomotic leakage occur simultaneously, the condition can rapidly become serious and life-threatening in patients with cirrhosis. Staged operation could certainly reduce the possibility of simultaneous anastomotic failure and pulmonary complications, because resection and reconstruction are performed separately. We believe that staged operation should be considered in esophageal carcinosarcoma patients with liver cirrhosis in order to avoid serious conditions after esophagectomy.

Because blind dissection is required to make a retrosternal route, which may cause uncontrollable bleeding, surgeons should avoid this procedure in patients with underlying liver cirrhosis patients. The subcutaneous route may be safe for reconstruction, because anastomotic leakage, which is especially frequent in patients with liver cirrhosis, is unlikely to become fatal.

Esophageal cancer patients sometimes have liver cirrhosis despite showing almost normal results of blood tests; therefore, it is important for surgeons to have a high index of suspicion based on the history and imaging findings. In our case reported here, the surgical invasiveness could be reduced, as the patient was diagnosed as having liver cirrhosis prior to the esophagectomy.

## 4 Conclusion

Although liver cirrhosis patients undergoing esophagectomy are at a high risk for hospital mortality, staged operation could potentially manage esophageal carcinosarcoma patient coexisting with liver cirrhosis.

## Ethical approval

Ethical approval exemption was given for this study.

## Consent

Consent to publish was obtained from this patient, and the identity of this patient was protected.

## Funding

The authors declare that they received no funding support for this report.

## Author contribution

FK reviewed the literature and wrote the manuscript. KK was the patient's surgeon, reviewed the literature, and contributed to manuscript drafting. SS provided the information on the histopathology. TN and KH were the patient's surgeons and were involved in the perioperative management. JS contributed to drafting of the manuscript. all authors issued final approval for this version of the manuscript to be submitted.

## Guarantor

Kazuo Koyanagi, corresponding author of this article.

## Research registration number

Not applicable.

## Declaration of competing interest

The authors report no declarations of interest.
